# Dynamic Determinants of Longevity and Exceptional Health

**DOI:** 10.1155/2010/381637

**Published:** 2010-09-30

**Authors:** Anatoli I. Yashin, Konstantin G. Arbeev, Igor Akushevich, Liubov Arbeeva, Julia Kravchenko, Dora Il'yasova, Alexander Kulminski, Lucy Akushevich, Irina Culminskaya, Deqing Wu, Svetlana V. Ukraintseva

**Affiliations:** ^1^Center for Population Health and Aging, Duke University, Durham, NC 27708, USA; ^2^Duke Comprehensive Cancer Center, Durham, NC 27705, USA

## Abstract

It is well known from epidemiology that values of indices describing physiological state in a given age may influence human morbidity and mortality risks. Studies of connection between aging and life span suggest a possibility that dynamic properties of age trajectories of the physiological indices could also be important contributors to morbidity and mortality risks. In this paper we use data on longitudinal changes in body mass index, diastolic blood pressure, pulse pressure, pulse rate, blood glucose, hematocrit, and serum cholesterol in the Framingham Heart Study participants, to investigate this possibility in depth. We found that some of the variables describing individual dynamics of the age-associated changes in physiological indices influence human longevity and exceptional health more substantially than the variables describing physiological state. These newly identified variables are promising targets for prevention aiming to postpone onsets of common elderly diseases and increase longevity.

## 1. Introduction

**Figure 1:**
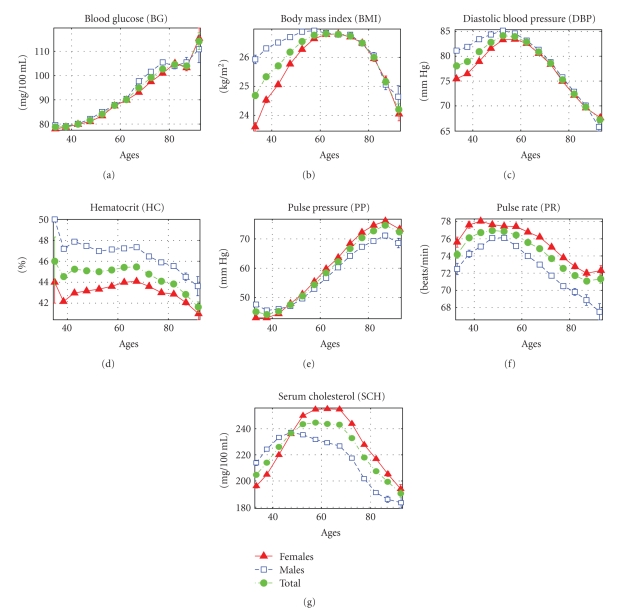
Mean values (± s.e.) of physiological indices in participants of the original cohort of the Framingham Heart Study (pooled data of available measurements from exams 1–25).

Individual age trajectories of physiological indices result from complicated interplay among genetic and environmental (including behavioral) factors taking place during the aging process and so, they may differ substantially among individuals in cohort. Despite this fact the average age trajectories for the same index follow remarkable regularities.  Figure 1 shows the average age trajectories of selected physiological indices evaluated from the data on the original cohort of the Framingham Heart Study (FHS). 

One can see from this figure that some indices tend to change monotonically with age: the level of blood glucose (BG) increases almost monotonically; the pulse pressure (PP) increases from age 40 till age 85, then levels off and shows a tendency to decline only at later ages. The age trajectories of other indices are nonmonotonic: they tend to increase first and then decline. Physiological average body mass index (BMI) increases up to about age 70 and then declines, diastolic blood pressure (DBP) increases till age 55–60 and then declines, serum cholesterol (SCH) increases till age 50 in males, and till age 70 in females and then declines, pulse rate (PR) increases till age 55 in males and till age 45 in females and then declines, hematocrit (HC) declines after age 70 in both sexes. With small variations, these general patterns are similar in males and females. 

The effects of these indices on mortality risk were studied in [1–3]. It was found that these effects are gender and age specific. The fact that the age dependence affects the shape of mortality risk function provided important insights into the mechanisms by which aging process affects the decline in stress resistance in individuals [4–6]. It was also found that dynamic properties of individual age trajectories of physiological indices may differ dramatically from one individual to the next. 

Researchers continue the debates about determinants of the aging rate and about possible contribution of this rate to life span and healthy life span [7–17]. Since the rate of aging literally means the rate of changing with age, it would be reasonable to assume that individual differences in the aging rate are to be manifested in variability of dynamic properties of individual age trajectories of physiological indices. And if individual aging rate affects life span and healthy life span then one can expect that dynamic characteristics of such trajectories will affect morbidity and mortality risks. 

A number of studies available in the literature support the view about the importance of using dynamic properties of individual age associated changes in physiological indices as the characteristics of aging process that predict morbidity and mortality risks, in addition to the use of the age-specific baseline measurements [18–24].

In this paper we investigate the effects of selected parameters describing the dynamic properties of the age trajectories of seven physiological indices on consequent morbidity and mortality risks in participants of the FHS original cohort. 

## 2. Data and Method

### 2.1. The Framingham Heart Study (FHS)

The FHS Original cohort was launched in 1948 (Exam 1), with 5,209 respondents (55% females) aged 28–62 years residing in Framingham, Massachusetts, who had not yet developed overt symptoms of cardiovascular disease, and continued to the present with biennial examinations (29 exams to date, data from exams 1–25 were used in this study) that include detailed medical history, physical exams, and laboratory tests.

### 2.2. Phenotypic Traits

Phenotypic traits collected in the FHS cohorts over 60 years and relevant to our analyses include life span, ages at onset of diseases (with the emphasis on cardiovascular diseases (CVD), cancer, and diabetes mellitus), as well as indices characterizing physiological state. The occurrence of diseases (CVD and cancer) and death has been followed through continuous surveillance of hospital admissions, death registries, clinical exams, and other sources, so that all the respective events are included in the study. We used data on onset of CVD, cancer (calculated from the followup data) and diabetes (defined as the age at the first exam when an individual has a value of BG exceeding 140 mg/dl and/or takes insulin and/or oral hypoglycemic agent) to define the age at onset of “unhealthy life” as the minimum of ages at onset of these three diseases. If an individual did not contract any of these diseases during the observation period than the individual was considered censored at the age of the last followup or death. Individuals who had any of the diseases before the first FHS exam were excluded from the analyses of “unhealthy life.” Data on physiological indices include random blood glucose (BG, exams 1–4, 6, 8–10, 13–23), body mass index (BMI, exams 1–25), diastolic blood pressure (DBP, exams 1–25), hematocrit (HC, exams 4–21), pulse pressure (PP, exams 1–25), pulse rate (PR, exams 1, 4–25), and serum cholesterol (SCH, exams 1–11, 13–15, 20, 22–25).

### 2.3. Definitions of “Dynamic” Risk Factors

We investigated dynamic properties of individual age trajectories of seven physiological indices mentioned above to select factors (referred to as “dynamic” risk factors) capable of affecting mortality risk and risk of onset of “unhealthy life.” BG was excluded from the list of indices for analyses of onset of “unhealthy life” because in the FHS data the onset of diabetes is specifically defined from the values of BG.

First, we evaluated the effect of the rate of changes in physiological indices at ages 40–60 on mortality risk and risk of onset of “unhealthy life” at ages 60+. For this purpose, we approximated the individual trajectories of those physiological indices that have a nearly linear dynamics (both for females and males) at ages 40–60 (BG, BMI, HC, and PP) by a linear function of the form *y*(*x*) = *a*_40−60_ + *b*_40−60_(*x* − 40), where *x* is age and *y* is the value of a physiological index at age *x*. Individuals having less than 5 observations of respective index at ages 40–60 were excluded from the analyses. As a result, we have estimates of three risk factors for each individual and each index: an initial value of an index at age 40 (i.e., *a*_40−60_, referred to as “*I* *n* *t* *e* *r* *c* *e* *p* *t*_40−60_” in Tables 1 and 2 and the text below), the rate of change in the physiological index at ages 40–60 (*b*_40−60_, “*S* *l* *o* *p* *e*_40−60_”), and the mean of absolute values of residuals, that is, deviations of observed values of an index from those approximated by a linear function at ages 40–60 (“*V* *a* *r* *i* *a* *b* *i* *l* *i* *t* *y*_40−60_”). The joint effect of these risk factors on mortality and incidence of “unhealthy life” was estimated (separately for each physiological index) by the Cox proportional hazards model with delayed entry (the left truncation time was defined as the maximum of the age at the first FHS exam and 60). Respectively, individuals with ages at death (onset of “unhealthy life”)/censoring below 60 were excluded from the analyses. Note that although the use of linear functions for describing individual aging-related changes is a rough approximation of monotonic changes, it captures important dynamic risk factor—the average rate of change of individual index at the age interval between 40 and 60 years.

**Figure 2:**
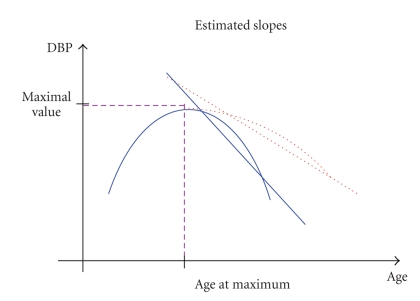
Dynamic characteristics of a hypothetical non-monotonically changing physiological index (denoted here “DBP”) considered as potential risk factors: 1) Maximum value; 2) Age at which the maximum has been reached; 3) Average rate of decline after reaching the maximum. The figure illustrates evaluation of average rates of decline in two individuals having the same pattern of increase until reaching the maximum and different patterns of decline after reaching the maximum: a) the solid line for a rapidly declining index and its approximation by a straight line; b) the dotted line for a slowly declining index and its linear approximation. The slopes of respective straight lines are considered as risk factors for mortality and onset of “unhealthy life.”

Second, we evaluated the effect of dynamic characteristics of physiological indices with nonmonotonic age trajectories on mortality risk and risk of onset of “unhealthy life.” For this purpose, we approximated the age trajectories of such indices (BMI, DBP, HC, PR, and SCH) by two linear functions. The first one approximates the increase in the trajectory at the initial interval [*x*_*L*_, *x*_max_]: *y*(*x*) = *a*_*L*_ + *b*_*L*_(*x* − *x*_*L*_), where *x* is age and *y* is the value of a physiological index at age *x*. The second one approximates the subsequent decline in the trajectory at the interval [*x*_max_, *x*_*R*_] after reaching the maximum value *y*_max_ = *a*_*L*_ + *b*_*L*_(*x*_max_ − *x*_*L*_) at age *x*_max_: *y*(*x*) = *a*_*R*_ + *b*_*R*_(*x* − *x*_max_). The intervals [*x*_*L*_, *x*_*R*_] for the fit were defined empirically for each index and sex as follows: [35, 55] for PR (females); [40, 60] for PR (males) and SCH (males); [45, 65] for BMI (males) and DBP (females and males); [50, 70] for SCH (females); [55, 75] for BMI (females) and HC (females and males). Note that the following restrictions on parameters were used in the estimation procedures: *b*_*L*_ > 0, *b*_*R*_ < 0, and *a*_*R*_ = *a*_*L*_ + *b*_*L*_(*x*_max_ − *x*_*L*_) to ensure the appropriate shape of the fit. Individuals having less than 6 observations of respective index at ages [*x*_*L*_ − 5, *x*_*R*_ + 5] and those having estimates of *b*_*L*_, *b*_*R*_ at the boundary of allowable values (i.e., nearly zero) were excluded from the analyses. As a result, we have estimates of six risk factors for each individual and each index: an initial value of an index at age *x*_*L*_ (i.e., *a*_*L*_, referred to as “*I* *n* *t* *e* *r* *c* *e* *p* *t*_2L_” in Tables 3 and 4 and the text below), the rate of increase in the physiological index at ages [*x*_*L*_, *x*_max_] (*b*_*L*_, “*Left Slope*”), the maximal value of the index approximated by two linear functions describing increase and decline in respective individual indices (*y*_max_, “*Max Index*”), age at reaching the maximal value of the index (*x*_max_, “*Age Max*”), the rate of decline in the index at ages [*x*_max_, *x*_*R*_] (*b*_*R*_, “*Right Slope*,” see also Figure 2 for illustration), and the mean of absolute values of residuals, that is, deviations of observed values of an index from those approximated by two linear functions at ages [*x*_*L*_, *x*_*R*_] (“*V* *a* *r* *i* *a* *b* *i* *l* *i* *t* *y*_2L_”). The joint effect of these risk factors on mortality and incidence of “unhealthy life” was estimated (separately for each physiological index) by the Cox proportional hazards model with delayed entry (the left truncation time was defined as the maximum of the age at the first FHS exam and *x*_*R*_). Respectively, individuals with ages at death (onset of “unhealthy life”)/censoring below *x*_*R*_ were excluded from the analyses. If *x*_*R*_ is different for females and males for some index, then the maximum of the two values was used in the (sex-adjusted) model applied to that index. Note that all these calculations were performed for individual age trajectories of respective indices. As a result, each individual is now characterized by a vector of dynamic parameters.

**Figure 3:**
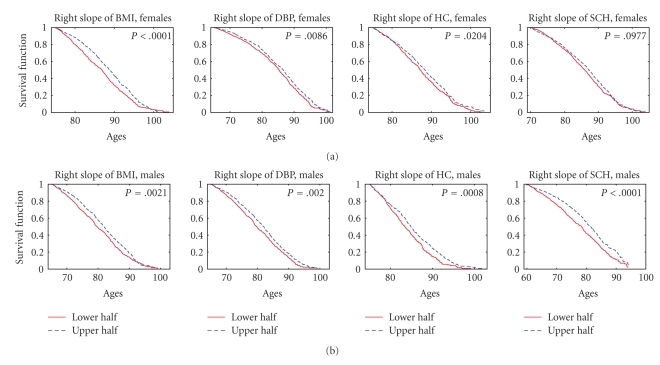
Kaplan-Meier estimates of survival functions for females (a) and males (b) having the average rate of decline of different physiological indices after reaching the maximum (“right slope,” see Section 2) from the lower and upper halves of empirical distributions of this risk factor for respective indices; *P* denotes *P *value for the null hypotheses about the equality of the survival curves in the strata evaluated by the log-rank test.

**Figure 4:**
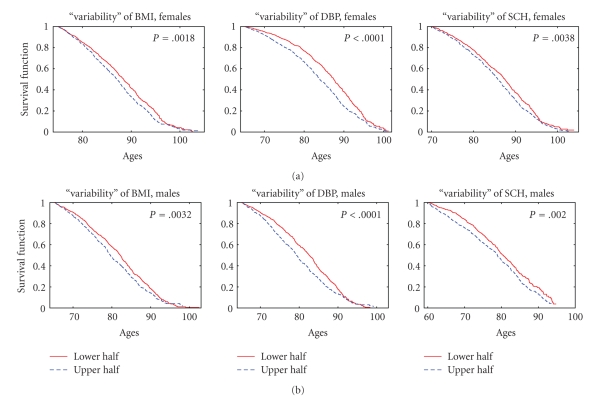
Kaplan-Meier estimates of survival functions for females (a) and males (b) having “variability” of different physiological indices (the mean of absolute values of residuals, i.e., deviations of observed values of an index from those approximated by two linear functions at respective age intervals, see Section 2) from the lower and upper halves of empirical distributions of this risk factor for respective indices; *P* denotes *P *value for the null hypotheses about the equality of the survival curves in the strata evaluated by the log-rank test.

**Figure 5:**
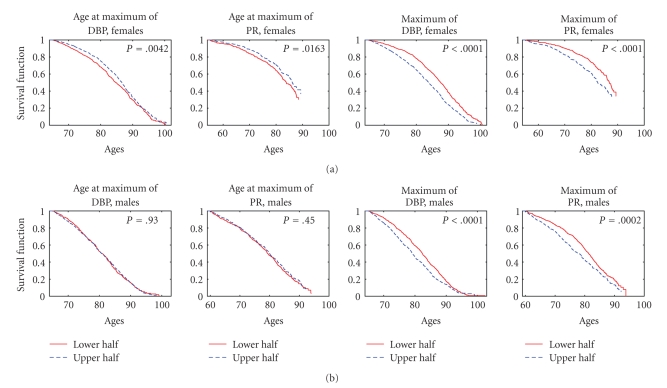
Kaplan-Meier estimates of survival functions for females (a) and males (b) having ages at reaching the maximum and the estimated maximal value (see Section 2) of different physiological indices from the lower and upper halves of empirical distributions of these risk factors for respective indices; *P* denotes *P *value for the null hypotheses about the equality of the survival curves in the strata evaluated by the log-rank test.

**Figure 6:**
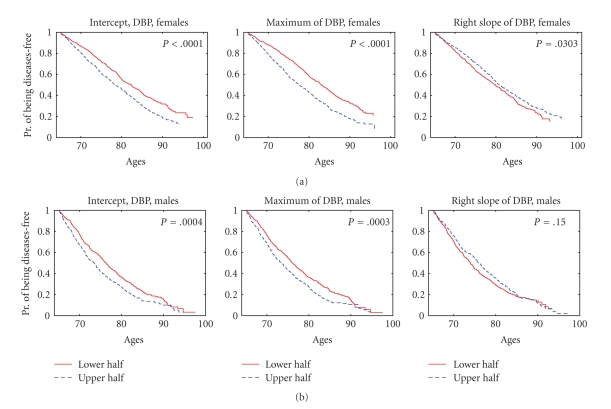
Kaplan-Meier estimates of probabilities of staying free of the diseases defining the onset of “unhealthy life” for females (a) and males (b) having initial values of diastolic blood pressure (DBP) at age 65, the estimated maximal values of DBP, and the average rates of decline of DBP after reaching the maximum (“intercept,” “maximum,” and “right slope,” respectively, see Section 2) from the lower and upper halves of empirical distributions of these risk factors; *P* denotes *P *value for the null hypotheses about the equality of the survival curves in the strata evaluated by the log-rank test.

We also evaluated the empirical (Kaplan-Meier) estimates of survival functions (and probabilities of staying free of the diseases defining the onset of “unhealthy life”) for individuals with different values of the dynamic risk factors based on the indices with nonmonotonic trajectories (separately for females and males). For each physiological index and each dynamic risk factor (“*Age Max,*” “*Max Index,*” “*I* *n* *t* *e* *r* *c* *e* *p* *t*_2L_,” “*Left Slope,*” “*Right Slope,*” and “*V* *a* *r* *i* *a* *b* *i* *l* *i* *t* *y*_2L_”), we calculated the values of the risk factor in all eligible individuals from the sample using the procedure described above. Then we evaluated the medians of such empirical distributions of risk factors, separately for females and males. These median values were used to define the sex-specific strata for estimation of survival curves. We assigned individuals of respective sex into one of two strata depending on whether the value of the risk factor for this individual is below (this stratum is denoted as “lower half” in Figures 3–6) or above (denoted as “upper half” in Figures 3–6) the (sex-specific) median value. In case of an odd number of individuals, the individual with the value of the risk factor equal to the median was assigned to the upper stratum. Then we calculated the Kaplan-Meier estimates of survival curves (conditional at the sex- and index-specific ages *x*_*R*_) for individuals in these two strata. Note that individuals with ages at death (onset of “unhealthy life”)/censoring below *x*_*R*_ were excluded from the analyses, as described above. Respective graphs are shown in Figures 3–6. For example, the median value of the right slopes calculated for BMI in females equals −0.103. Hence, individuals from the stratum denoted as “lower half” in the upper left graph of Figure 3 are females with values of the right slope of BMI smaller than −0.103. Individuals belonging to the stratum named “upper half” in the upper left graph of Figure 3 are females with values of the right slope of BMI larger than −0.103. The conditional survival curves for the two strata presented in this figure deal with individuals survived until age 75 years, which is the value of *x*_*R*_ for BMI in females, as described above.

### 2.4. Statistical Analyses

Statistical analyses and graphic output were performed with SAS/STAT (SAS Institute Inc.) and MATLAB (MathWorks Inc.) software packages. *P* values for the regression parameters in the tables were calculated using the Wald chi-square statistic with respect to a chi-square distribution with one degree of freedom using SAS/STAT PROC PHREG. The log-rank test was used to test the null hypotheses about the equality of the empirical survival curves in the strata. Respective *P* values are shown in Figures 3–6 (SAS/STAT PROC LIFETEST was used for these purposes).

## 3. Results

### 3.1. Effect of Individual Dynamics of Physiological Indices at Ages 40–60 on Mortality Risk and Risk of Onset of “Unhealthy Life” at Ages 60+

As described in Section 2, we evaluated the effect of individual dynamics of physiological indices at ages 40–60 on mortality risk and risk of onset of “unhealthy life” at ages 60+ for those indices that have a nearly linear pattern of change at the age interval 40–60 for both females and males. Table 1 shows the estimates of the joint effect of these risk factors on mortality as evaluated by the Cox proportional hazards model. One can see from this table that the variability around the average linear trajectory (“*V* *a* *r* *i* *a* *b* *i* *l* *i* *t* *y*_40−60_”) and the average rate of change between ages 40 and 60 (*“* *S* *l* *o* *p* *e*_40−60_”) are significant risk factors for mortality for all indices. The significance is highest (*P* < .0001) for the slopes of HC and PP. The initial value of an index at age 40 (“*I* *n* *t* *e* *r* *c* *e* *p* *t*_40−60_”) is also a highly significant (*P* < .0001) risk factor for mortality for HC and PP (i.e., higher values of respective index at age 40 correspond to higher risk of death compared to smaller values of this index), being nonsignificant for BG. 

The effect of these dynamic characteristics on incidence of “unhealthy life” is similar (see Table 2). However, the variability is significant only for PP. Note that the effect of variable *“Sex”* on both mortality and risk of onset of “unhealthy life” is significant and that the risk for males is higher than those for females.

### 3.2. Effect of Dynamic Characteristics of Physiological Indices with Nonmonotonic Age Trajectories on Mortality Risk and Risk of Onset of “Unhealthy Life”

For indices with nonmonotonic age trajectories, we evaluated the maximum value of respective index, age at which this maximum is reached, the intercept, and the left and right slopes of the linear functions approximating the increase and decline of respective indices as described in Section 2. Tables 3 and 4 show the estimates of the joint effect of these risk factors on mortality and incidence of “unhealthy life” as evaluated by the Cox proportional hazards model. One can see from Table 3 that the effect of the rate of decline in the index after reaching the maximum (“*Right Slope*”) on mortality risk is significant for all indices (the highest significance, *P* < .0001, is observed for BMI and DBP). In this case, the faster decline in the index after reaching the maximum corresponds to a significant increase in mortality risk (note that the values of “*Right Slope*” are negative by definition, see Section 2). On the contrary, the rate of increase in the index before reaching the maximum (“*Left Slope*”) and the initial value from which the increase has started (“*I* *n* *t* *e* *r* *c* *e* *p* *t*_2L_”) are not significant risk factors for mortality for any index. The estimated maximal value of the index reached is also a significant risk factor for mortality in case of DBP, PR (both have *P* < .0001), and HC (*P* < .05). This means that the larger (sex-adjusted) maximal values of respective indices (reached at *“Age Max”*) correspond to a significant increase in mortality risk. The age at reaching the maximum is itself a significant risk factor for mortality for these indices. The negative values of respective estimates indicate that the later an individual reaches the maximum, the smaller mortality risk is. The variability in all indices except PR significantly affects mortality risks (the larger variability corresponds to higher mortality risks).

The effect of these dynamic characteristics on risk of onset of “unhealthy life” is less pronounced than that on mortality risks. Table 4 shows that the rate of decline after reaching the maximum (“*Right Slope*”), age at reaching the maximum (“*Age Max*”), and the variability become nonsignificant for all indices. The maximal value reached is significant only for DBP (*P* < .0001) and PR (*P* < .05). The rate of increase of BMI before reaching the maximum becomes significant (*P* < .05), with the faster rate of increase corresponding to a higher risk of onset of “unhealthy life.” Note again that the effect of variable “*Sex*” on both mortality and risk of onset of “unhealthy life” is significant and that the risk for males is higher than that for females.

### 3.3. Effect of Dichotomized Dynamic Characteristics of Physiological Indices with Nonmonotonic Age Trajectories

We also evaluated the Kaplan-Meier estimates of survival functions for individuals with different values of the dynamic risk factors based on the indices with nonmonotonic trajectories dividing the entire sex-specific samples into strata representing individuals with the values of the index in the lower and upper halves of the empirical distribution of respective index (see Section 2). 

Figure 3 shows the estimates of survival functions for females and males having the average rate of decline of different physiological indices after reaching the maximum (“*Right Slope*”) from the lower and upper halves of empirical distributions of this risk factor for respective indices. One can see from this figure that the lower absolute values of the slope (i.e., the lower rates of the postmaximum decline) in individuals from the upper half of the distribution are associated with better survival for all indices except SCH for females (nonsignificant results for PR for both sexes are not shown). The highest significance (*P* < .0001) is observed for BMI in females and SCH in males.

Figure 4 illustrates similar estimates in case of “variability” of different physiological indices (the mean of absolute values of residuals, that is, deviations of observed values of an index from those approximated by two linear functions at respective age intervals, see Section 2). The higher “variability” of trajectories of BMI, DBP, and SCH at respective age intervals in individuals from the upper half of the distribution result in worse survival for both females and males (nonsignificant results for HC and PR are not shown). The highest significance (*P* < .0001) is observed for DBP in both females and males.

Later ages at reaching the maximal value of DBP and PR in females from the upper half of the distribution are associated with better survival (Figure 5), however this was not observed for males. The higher estimated maximal values of these indices in individuals from the upper half of the distribution correspond to worse survival for both females and males. All other indices did not produce any significant results and are not shown in Figure 5.

Similar calculations for probabilities of staying free of the diseases defining the onset of “unhealthy life” revealed a more mosaic picture. The most consistent results were observed for DBP (Figure 6). 

The higher initial values of DBP at age 45 and the higher estimated values of DBP reached in individuals from the upper halves of respective distributions are associated with worse chances of staying free of the “unhealthy life,” for both sexes. The lower rates of the postmaximum decline of DBP in females, but not males, from the upper half of the distribution correspond to better chances of staying free of the “unhealthy life” (Figure 6). In addition, earlier ages at reaching the maximum of SCH for females (*P* = .008), the smaller estimated maximal values of BMI (*P* = .005) and HC (*P* = .02) for females, and PR (*P* = .001) and SCH (*P* = .017) for males, and a smaller initial value of BMI at age 55 (*P* = .001) for females and a smaller initial value of SCH at age 40 (*P* = .0004) for males, were related to better chances of staying free of the “unhealthy life.”

### 3.4. Sensitivity Analyses

We should note that the question about the effect of the quality of estimates is important given that at most 11 observations for the monotone indices or 15 observations for non-monotone indices were used (note that for non-monotone indices data from 30-year intervals [**x**_**L**_ − 5, *x*_*R*_ + 5], where *x*_*R*_ − **x**_**L**_ = 20, were used for calculating dynamic risk factors). These observations were used to estimate two parameters (those of the linear regression) for monotone indices and four parameters (age at reaching the maximal value of the index, intercept, and two slopes) for non-monotone indices. To partly reduce the effect of a poor fit due to a small number of longitudinal observations, we removed those individuals having less than 5 (less than 6 for non-monotone indices) observations from analyses. Clearly, the results could change had we used different numbers for the minimal allowable numbers of observations. To test how sensitive our results are to such changes, we performed sensitivity analyses with different minimal allowable numbers of observations: 4, 6, and 7 for monotone indices, and 5, 7, and 8 for non-monotone ones. The results showed that the effect of dynamic risk factors calculated from individual trajectories of monotone indices at ages 40–60 on mortality risk and risk of onset of “unhealthy life” at ages 60+ is stable across different studies. All risk factors for which the estimates of the regression parameters were significant (*P* < .01) in the original study, exhibited similar significant effects on mortality risk or risk of onset of “unhealthy life” in the other studies. Despite some variability in the values of the estimates across the studies, the “direction” of the effect (i.e., the sign of the estimate) was the same in all such cases. In the sensitivity analysis with the largest cutoff value, the *P* values were somewhat larger in some cases which may be explained by a smaller sample size compared to the original study. The effects of dynamic risk factors calculated from individual trajectories of physiological indices with nonmonotonic patterns on mortality risk and risk of onset of “unhealthy life” showed similar stability across the studies as the monotone indices. In all cases (except for the right slope of PR which appeared either marginally significant or nonsignificant in the other studies), risk factors for which the estimates of the regression parameters were significant (*P* < .01) in the original study exhibited similar significant effects on mortality risk or risk of onset of “unhealthy life” in other studies. The same observation regarding the sensitivity analysis based on the largest cutoff value was true for non-monotone risk factors too.

## 4. Discussion

An increase in mortality rate with age is traditionally associated with progressing aging. This influence is mediated by the aging-associated changes in thousands of biological and physiological variables, some of which have been measured in aging studies. The fact that the age trajectories of some of such variables differ among individuals with short and long life spans and healthy life spans indicates that dynamic properties of respective indices affect the life history traits. Our analyses of the FHS data clearly demonstrate that the values of physiological indices at age 40 are significant contributors to both life span and healthy life span (as show the estimates of *I* *n* *t* *e* *r* *c* *e* *p* *t*_40−60_ in Tables 1 and 2), suggesting that normalizing these variables around the age 40 is important for preventing age-associated morbidity and mortality later in life. Two dynamic parameters, *S* *l* *o* *p* *e*_40−60_ and *V* *a* *r* *i* *a* *b* *i* *l* *i* *t* *y*_40−60_, also have significant effect on mortality risk (the former being more important predictor in case of healthy life span). These data suggest that keeping physiological indices stable over the years of life could be as important as their normalizing around the age 40. Thus, a slower change in an index with age is likely to indicate the slower aging and the lower morbidity and mortality risks.

Table 3 shows that dynamic properties of the indices that change nonmonotonically with age significantly contribute to mortality risks and further demonstrates the importance of maintaining stability of physiological state in aging humans: the lower rate of decline in an index after reaching the age at maximum means the more beneficial effect on all-cause mortality.

The fact that the effect of the studied dynamic characteristics on risks of “unhealthy life” onset (Table 4) is less pronounced than that on all-cause mortality risk may indicate that the dynamic characteristics reflect basic aging-related processes in body that result in increasing nonspecific vulnerability to death with age rather than in increasing vulnerability to a particular pathology.

The review of the literature (below) supports our findings with respect to importance of taking into account longitudinal changes in physiological indices when evaluating/predicting morbidity and mortality risks. One should note, however, that the impact and comparative contributions of dynamic parameters (left and right slopes, variability, intercept) on mortality risks were evaluated in our study for the first time. In our two recent publications we demonstrated that individuals who have different rates of aging related changes in BG levels also differ in longevity [2, 3]. 

The effects of aging associated changes in serum cholesterol on coronary and all-cause mortality were evaluated in Finnish Cohorts of the Seven Countries study [22]. Men with greatest declines in the cholesterol levels had increased cardiovascular and all-cause mortality compared with men with least change in the levels. In the Paris Prospective Study [23], it was shown that not only a low baseline total cholesterol level but also its decline over time was associated with a higher cancer mortality. 

Similar to SCH, high blood pressure (BP) is a major risk factor for CVD. A study of two independent French male cohorts suggested that longitudinal changes in systolic and diastolic BP may be more accurate determinants of cardiovascular risks than baseline BP measures. In both cohorts, the group with a long-term increase in systolic and a decrease in diastolic BP (i.e., with increase in pulse pressure) had the highest relative risk of mortality from CVD compared to the group with no changes in either systolic or diastolic BP, independently of absolute values of BP or other risk factors [25]. Since this study included only males, it is important to note that changes in pulse pressure may in principle have different effects on mortality risks in males and females [26, 27]. 

The heart rate (HR) is one more index characterizing functioning of cardiovascular system. Prognostic importance of its baseline values as well as variability during 24-hour HR monitoring in patients with heart disease and in general population is recognized [28–30]. Contrarily, the prognostic role of the long-term and age-related dynamics of HR is not sufficiently investigated and respective studies are limited. A recent study of the effects of HR at baseline, final HR, and HR change during followup, on survival of patients attending the Glasgow Blood Pressure Clinic revealed that the highest risk of all-cause mortality was in patients who had increased their HR by ≥5 bpm at the end of followup, as compared with those who had a consistently high (high-high) or low (low-low) HR. Authors concluded that change in HR during the followup is a better predictor of mortality risk in hypertensive patients than baseline or final HR [31]. 

The body mass index (BMI) is, probably, the most intensively studied index in connection with health and survival. Over recent decades, many studies addressed the effect of BMI dynamics on morbidity and mortality, especially the effect of losing body weight in overweight/obese individuals on risk factors for CVD and diabetes. It was shown that overweight adults who lost weight over 9 years had more favorable (lower) total and LDL cholesterol levels compared to normal-weight control, but less favorable BG levels [32]. In other studies weight loss was associated with *excess* mortality when compared with weight stability, even when controlled for confounding due to diseases known to cause both weight loss and increased mortality [33, 34].

It was also shown that the weight stability was associated with a lower mortality risk as compared with weight change (gain or loss) [35, 36]. Nilsson et al. [37], showed that in men with decreasing BMI during 16 years of followup the noncancer mortality was higher compared to BMI-stable men. Authors hypothesized that involuntary weight loss in otherwise healthy people could be a sign of premature aging, which in turn caused a nonspecific increase in mortality risk. In other studies, baseline weight and weight change had independent effects on total mortality, with both the associations exhibiting a U-shaped relation [38, 39]. 

Note that the seven physiological indices used in this paper do not exhaust the list of all possible physiological risk factors for mortality and morbidity. Therefore, the dynamic characteristics calculated from these seven indices cannot explain the entire variability in human life span and healthy life span. Other indices and risk factors can be explored on their association with mortality/morbidity risk if measurements of such indices are available in a longitudinal study for a substantially long-time period. See for example [40] where midlife risk factors were investigated for a cohort of Japanese American men with 40 years follow up.

## 5. Conclusion

In sum, our results indicate that the dynamic characteristics of age-related changes in physiological variables are important predictors of morbidity and mortality risks in the aging individuals. Previously published epidemiological findings are generally in concert with our results, which clearly indicates the need for further detailed studies of the dynamic parameters of aging related changes in human body with further application of these principles to the prevention strategies. We showed that the rate of changes in physiological state at the age interval between 40 and 60 years may serve as a good predictor of morbidity and mortality risks later in life. For nonmonotonically changing indices, the rates of decline after reaching the maximum, the maximal values, and the age at the maximum are important predictors of morbidity and mortality risks. 

Senescence is likely to be the key player in physiological and biological changes observed in aging humans. The dynamic properties of these changes contain important information about the individual aging processes. This information, however, can be masked by the effects of compensatory adaptation and remodeling developing in response to the primary aging process. Studying mechanisms of such adaptation and its connection to morbidity and mortality risks is important for better understanding factors and mechanisms affecting long and healthy life.

## Figures and Tables

**Table 1:** Effect of “dynamic” risk factors calculated from individual trajectories of physiological indices at ages 40–60 on mortality risk at ages 60+ in the Framingham Heart Study (original cohort) estimated by the Cox proportional hazards model.

Physiological Index	Risk Factor (RF)	Mean RF (St. Dev.)	Cox Regression Model	
			Parameter (S.E.)	Hazard Ratio (95% C.I.)
BG (*N* = 2224, *N*_*e*_ = 1447, *N*_*c*_ = 777)	Intercept_40−60_	77.468 (20.370)	0.003 (0.002)	1.056 (0.978, 1.140)
	Slope_40−60_	0.553 (1.932)	0.059* (0.029)	1.088 (1.002, 1.182)
	Variability_40−60_	8.518 (6.798)	0.017^#^ (0.005)	1.086 (1.033, 1.141)
	Sex		0.581^†^ (0.053)	1.789 (1.611, 1.985)

BMI (*N* = 3150, *N*_*e*_ = 2217, *N*_*c*_ = 933)	Intercept_40−60_	25.867 (4.215)	0.016^#^ (0.006)	1.086 (1.020, 1.157)
	Slope_40−60_	0.050 (0.171)	−0.305* (0.141)	0.945 (0.897, 0.995)
	Variability_40−60_	0.697 (0.392)	0.176^#^ (0.060)	1.074 (1.024, 1.126)
	Sex		0.564^†^ (0.045)	1.757 (1.610, 1.918)

HC (*N* = 2167, *N*_*e*_ = 1323, *N*_*c*_ = 844)	Intercept_40−60_	45.341 (4.664)	0.086^†^ (0.011)	1.622 (1.430, 1.839)
	Slope_40−60_	−0.026 (0.272)	0.932^†^ (0.172)	1.311 (1.189, 1.446)
	Variability_40−60_	1.548 (0.633)	0.089* (0.044)	1.073 (1.002, 1.148)
	Sex		0.255^§^ (0.071)	1.291 (1.123, 1.484)

PP (*N* = 3153, *N*_*e*_ = 2219, *N*_*c*_ = 934)	Intercept_40−60_	44.112 (13.095)	0.024^†^ (0.002)	1.349 (1.273, 1.428)
	Slope_40−60_	0.506 (0.846)	0.338^†^ (0.035)	1.360 (1.277, 1.448)
	Variability_40−60_	4.815 (2.032)	0.036^#^ (0.012)	1.090 (1.033, 1.150)
	Sex		0.611^†^ (0.044)	1.842 (1.691, 2.007)

Notes. *.01 ≤ *P* < .05, ^#^.001 ≤ *P* < .01, ^§^.0001 ≤ *P* < .001, ^†^ *P* < .0001, for other estimates: *P* ≥ .05; *Sex*: 1—male, 0—female; the other *Risk Factors* are continuous and calculated as described in Section 2; *N* denotes the total number of individuals; *N*_*e*_ is the total number of events (deaths); *N*_*c*_ is the total number of censored individuals; *Hazard Ratios* for continuous risk factors are for an increase from the first quartile to the third quartile of respective empirical distributions.

**Table 2:** Effect of “dynamic” risk factors calculated from individual trajectories of physiological indices at ages 40–60 on risk of onset of “unhealthy life” at ages 60+ in the Framingham Heart Study (original cohort) estimated by the Cox proportional hazards model.

Physiological Index	Risk Factor (RF)	Mean RF (St. Dev.)	Cox Regression Model	
			Parameter (S.E.)	Hazard Ratio (95% C.I.)
BMI (*N* = 2458, *N*_*e*_ = 1824, *N*_*c*_ = 634)	Intercept_40−60_	25.587 (3.954)	0.036^†^ (0.007)	1.198 (1.119, 1.283)
	Slope_40−60_	0.057 (0.162)	0.609^§^ (0.159)	1.116 (1.055, 1.180)
	Variability_40−60_	0.679 (0.381)	0.013 (0.070)	1.005 (0.953, 1.060)
	Sex		0.511^†^ (0.049)	1.668 (1.513, 1.837)

HC (*N* = 1659, *N*_*e*_ = 1192, *N*_*c*_ = 467)	Intercept_40−60_	45.044 (4.641)	0.078^†^ (0.012)	1.556 (1.365, 1.774)
	Slope_40−60_	−0.021 (0.269)	1.140^†^ (0.182)	1.384 (1.250, 1.533)
	Variability_40−60_	1.547 (0.635)	0.082 (0.046)	1.069 (0.993, 1.150)
	Sex		0.287^†^ (0.073)	1.332 (1.155, 1.536)

PP (*N* = 2460, *N*_*e*_ = 1825, *N*_*c*_ = 635)	Intercept_40−60_	43.612 (12.635)	0.018^†^ (0.003)	1.249 (1.170, 1.334)
	Slope_40−60_	0.480 (0.814)	0.319^†^ (0.040)	1.325 (1.237, 1.420)
	Variability_40−60_	4.667 (1.944)	0.046^§^ (0.014)	1.111 (1.046, 1.181)
	Sex		0.577^†^ (0.048)	1.781 (1.622, 1.957)

Notes. *.01 ≤ *P* < .05, ^#^.001 ≤ *P* < .01, ^§^.0001 ≤ *P* < .001, ^†^ *P* < .0001, for other estimates: *P* ≥ .05; *Sex*: 1—male, 0—female; the other *Risk Factors* are continuous and calculated as described in Section 2; *N* denotes the total number of individuals; *N*_*e*_ is the total number of events (onset of “unhealthy life”); *N*_*c*_ is the total number of censored individuals; *Hazard Ratios* for continuous risk factors are for an increase from the first quartile to the third quartile of respective empirical distributions.

**Table 3:** Effect of “dynamic” risk factors calculated from individual trajectories of physiological indices with nonmonotonic patterns on mortality risk in the Framingham Heart Study (original cohort) estimated by the Cox proportional hazards model.

Physiological Index	Risk Factor (RF)	Mean RF (St. Dev.)	Cox Regression Model	
			Parameter (S.E.)	Hazard Ratio (95% C.I.)
BMI (*N* = 2686, *N*_*e*_ = 1824, *N*_*c*_ = 862)	Age Max	62.063 (8.762)	−0.001 (0.004)	0.983 (0.887, 1.089)
	Max Index	27.869 (4.392)	−0.001 (0.012)	0.997 (0.884, 1.124)
	Intercept_2L_	26.171 (4.187)	0.007 (0.012)	1.034 (0.925, 1.156)
	Left Slope	0.220 (0.505)	−0.017 (0.049)	0.996 (0.975, 1.018)
	Right Slope	−0.224 (0.576)	−0.177^†^ (0.025)	0.959 (0.948, 0.970)
	Variability_2L_	0.729 (0.371)	0.356^†^ (0.073)	1.153 (1.088, 1.221)
	Sex		0.561^†^ (0.064)	1.753 (1.545, 1.989)

DBP (*N* = 3133, *N*_*e*_ = 2242, *N*_*c*_ = 891)	Age Max	55.165 (6.973)	−0.008* (0.004)	0.903 (0.822, 0.992)
	Max Index	86.804 (10.465)	0.016^†^ (0.003)	1.245 (1.141, 1.358)
	Intercept_2L_	80.471 (12.962)	0.001 (0.003)	1.008 (0.942, 1.079)
	Left Slope	0.842 (1.439)	0.006 (0.021)	1.006 (0.966, 1.047)
	Right Slope	−0.988 (1.976)	−0.055^†^ (0.010)	0.939 (0.918, 0.961)
	Variability_2L_	3.984 (1.383)	0.096^†^ (0.016)	1.172 (1.114, 1.233)
	Sex		0.514^†^ (0.043)	1.671 (1.536, 1.818)

HC (*N* = 2471, *N*_*e*_ = 1650, *N*_*c*_ = 821)	Age Max	66.061 (7.020)	−0.009* (0.004)	0.882 (0.795, 0.978)
	Max Index	46.567 (3.265)	0.024* (0.012)	1.108 (1.003, 1.224)
	Intercept_2L_	43.756 (4.848)	0.007 (0.008)	1.031 (0.960, 1.108)
	Left Slope	0.390 (0.733)	−0.011 (0.054)	0.996 (0.956, 1.037)
	Right Slope	−0.856 (3.533)	−0.018* (0.007)	0.988 (0.979, 0.997)
	Variability_2L_	1.472 (0.551)	0.099* (0.045)	1.066 (1.006, 1.129)
	Sex		0.398^†^ (0.060)	1.488 (1.323, 1.675)

PR (*N* = 1847, *N*_*e*_ = 1097, *N*_*c*_ = 750)	Age Max	47.279 (7.676)	−0.012* (0.005)	0.851 (0.742, 0.977)
	Max Index	81.206 (10.689)	0.016^†^ (0.004)	1.247 (1.126, 1.381)
	Intercept_2L_	71.554 (15.226)	−0.002 (0.003)	0.979 (0.904, 1.059)
	Left Slope	1.535 (3.445)	0.007 (0.013)	1.011 (0.972, 1.052)
	Right Slope	−0.898 (1.980)	−0.070^§^ (0.021)	0.927 (0.886, 0.970)
	Variability_2L_	5.057 (1.978)	0.028 (0.017)	1.070 (0.988, 1.159)
	Sex		0.727^†^ (0.070)	2.069 (1.804, 2.374)

SCH (*N* = 2297, *N*_*e*_ = 1711, *N*_*c*_ = 586)	Age Max	55.574 (8.298)	0.002 (0.005)	1.023 (0.923, 1.134)
	Max Index	261.965 (42.429)	0.001 (0.001)	1.059 (0.958, 1.170)
	Intercept_2L_	225.428 (61.457)	−0.0003 (0.001)	0.981 (0.903, 1.066)
	Left Slope	5.517 (8.442)	−0.005 (0.005)	0.975 (0.921, 1.032)
	Right Slope	−4.121 (8.689)	−0.007* (0.003)	0.969 (0.946, 0.993)
	Variability_2L_	13.484 (6.237)	0.014^#^ (0.004)	1.101 (1.039, 1.166)
	Sex		0.566^†^ (0.073)	1.761 (1.526, 2.031)

Notes. *.01 ≤ *P* < .05, ^#^.001 ≤ *P* < .01, ^§^.0001 ≤ *P* < .001, ^†^ *P* < .0001, for other estimates: *P* ≥ .05; *Sex*: 1—male, 0—female; the other *Risk Factors* are continuous and calculated as described in Section 2; *N* denotes the total number of individuals; *N*_*e*_ is the total number of events (deaths); *N*_*c*_ is the total number of censored individuals; *Hazard Ratios* for continuous risk factors are for an increase from the first quartile to the third quartile of respective empirical distributions.

**Table 4:** Effect of “dynamic” risk factors calculated from individual trajectories of physiological indices with nonmonotonic patterns on risk of onset of “unhealthy life” in the Framingham Heart Study (original cohort) estimated by the Cox proportional hazards model.

Physiological Index	Risk Factor (RF)	Mean RF (St. Dev.)	iCox Regression Model	
			Parameter (S.E.)	Hazard Ratio (95% C.I.)
BMI (*N* = 1380, *N*_*e*_ = 782, *N*_*c*_ = 598)	Age Max	63.199 (8.535)	0.012 (0.006)	1.189 (0.997, 1.417)
	Max Index	27.383 (4.016)	−0.011 (0.024)	0.947 (0.757, 1.186)
	Intercept_2L_	25.696 (3.875)	0.037 (0.025)	1.184 (0.954, 1.469)
	Left Slope	0.194 (0.350)	0.340* (0.155)	1.073 (1.007, 1.143)
	Right Slope	−0.235 (0.801)	−0.041 (0.044)	0.991 (0.972, 1.011)
	Variability_2L_	0.703 (0.359)	−0.007 (0.121)	0.997 (0.910, 1.092)
	Sex		0.497^†^ (0.102)	1.644 (1.346, 2.009)

DBP (*N* = 2139, *N*_*e*_ = 1512, *N*_*c*_ = 627)	Age Max	55.325 (7.112)	−0.006 (0.004)	0.926 (0.827, 1.037)
	Max Index	85.806 (9.966)	0.017^†^ (0.004)	1.239 (1.129, 1.360)
	Intercept_2L_	79.309 (12.066)	0.001 (0.003)	1.013 (0.940, 1.093)
	Left Slope	0.912 (1.808)	0.009 (0.017)	1.008 (0.978, 1.040)
	Right Slope	−0.980 (2.390)	−0.014 (0.009)	0.986 (0.968, 1.004)
	Variability_2L_	3.850 (1.311)	0.039 (0.020)	1.064 (0.999, 1.133)
	Sex		0.488^†^ (0.053)	1.629 (1.470, 1.806)

HC (*N* = 1254, *N*_*e*_ = 705, *N*_*c*_ = 549)	Age Max	66.094 (7.125)	−0.011 (0.006)	0.863 (0.732, 1.017)
	Max Index	46.088 (3.141)	0.028 (0.018)	1.129 (0.967, 1.319)
	Intercept_2L_	43.417 (3.810)	−0.002 (0.016)	0.990 (0.868, 1.130)
	Left Slope	0.372 (0.713)	−0.041 (0.078)	0.985 (0.933, 1.041)
	Right Slope	−0.775 (2.966)	0.0002 (0.013)	1.000 (0.985, 1.016)
	Variability_2L_	1.442 (0.534)	0.033 (0.074)	1.021 (0.930, 1.122)
	Sex		0.363^†^ (0.091)	1.438 (1.203, 1.718)

PR (*N* = 1401, *N*_*e*_ = 1013, *N*_*c*_ = 388)	Age Max	47.167 (7.589)	0.004 (0.005)	1.057 (0.921, 1.213)
	Max Index	80.449 (10.535)	0.009* (0.004)	1.129 (1.011, 1.262)
	Intercept_2L_	71.162 (13.289)	−0.001 (0.004)	0.987 (0.893, 1.091)
	Left Slope	1.443 (3.034)	0.007 (0.013)	1.010 (0.973, 1.049)
	Right Slope	−0.850 (1.650)	0.013 (0.023)	1.014 (0.968, 1.062)
	Variability_2L_	4.954 (1.936)	−0.002 (0.018)	0.994 (0.913, 1.082)
	Sex		0.569^†^ (0.070)	1.766 (1.539, 2.025)

SCH (*N* = 1361, *N*_*e*_ = 913, *N*_*c*_ = 448)	Age Max	56.821 (8.200)	0.005 (0.006)	1.063 (0.908, 1.245)
	Max Index	261.843 (42.286)	0.002 (0.001)	1.120 (0.978, 1.282)
	Intercept_2L_	225.919 (62.186)	−0.001 (0.001)	0.943 (0.854, 1.040)
	Left Slope	5.147 (8.013)	0.002 (0.007)	1.012 (0.936, 1.095)
	Right Slope	−4.329 (9.443)	−0.004 (0.004)	0.984 (0.952, 1.017)
	Variability_2L_	13.452 (6.246)	0.010 (0.006)	1.070 (0.986, 1.161)
	Sex		0.595^†^ (0.097)	1.813 (1.499, 2.192)

Notes. *0.01 ≤ *P* < .05, ^#^.001 ≤ *P* < .01, ^§^.0001 ≤ *P* < .001, ^†^ *P* < .0001, for other estimates: *P* ≥ .05; *Sex:* 1—male, 0—female; the other *Risk Factors* are continuous and calculated as described in Section 2; *N* denotes the total number of individuals; *N*_*e*_ is the total number of events (onset of “unhealthy life”); *N*_*c*_ is the total number of censored individuals; *Hazard Ratios* for continuous risk factors are for an increase from the first quartile to the third quartile of respective empirical distributions.
